# Identification of Workers at Increased Risk of Infection During a COVID-19 Outbreak in a Meat Processing Plant, France, May 2020

**DOI:** 10.1007/s12560-021-09500-1

**Published:** 2021-10-16

**Authors:** Yoann Mallet, Mathilde Pivette, Matthieu Revest, Elisabeth Angot, Marion Valence, Clarisse Dupin, Nicolas Picard, Guillaume Brelivet, Thomas Seyler, Stéphany Ballet, Alain Le Tertre, Yvonnick Guillois

**Affiliations:** 1 Regions Department, Santé Publique France (SpFrance), The French National Public Health Agency, Rennes, France; 2 Pontchaillou University Hospital, Infectious Diseases and Intensive Care Unit, and Rennes University, Inserm U1230, Rennes, France; 3 Department of Internal Medicine and Infectious Diseases, Yves Le Foll Hospital, Saint-Brieuc, France; 4 Biology Laboratory, Yves Le Foll Hospital, Saint-Brieuc, France; 5 Emergency Medical Aid Service (SAMU), Yves Le Foll Hospital, Saint-Brieuc, France; 6 Public Health Department, ARS Bretagne, The Breton Health Authorities, Rennes, France; 7 Alert and Crisis Department, Santé Publique France (SpFrance), Saint-Maurice, France; 8 Cellule Bretagne, Santé Publique France, C/O ARS Bretagne, 6 Place des Colombes, CS 14253, 35042 Rennes Cedex, France

**Keywords:** COVID-19, SARS-CoV-2, Outbreak, Meat plant

## Abstract

On 13 May 2020, a COVID-19 cluster was detected in a French processing plant. Infected workers were described. The associations between the SARS-CoV-2 infection and the socio-demographic and occupational characteristics were assessed in order to implement risk management measures targeting workers at increased risk of contamination. Workers were tested by RT-PCR from samples taken during screening campaigns. Workers who tested positive were isolated and their contacts were quarantined. Workers were described and associations with the SARS-CoV-2 infection were assessed through risk ratios using multivariable Poisson regression. Of the 1347 workers, 87.5% were tested: 140 cases were identified; 4 were hospitalised, including 2 admitted to intensive care. In the company, the cluster remained limited to deboning and cutting activities. The attack rate was 11.9% in the company, reaching 16.6% in the cutting department. Being an employee of a subcontractor significantly increased the risk of infection by 2.98 [1.81–4.99]. In the cutting department, an association with virus infection was found for a group of non-French speaking workers from the same Eastern European country (RR = 2.67 [1.76–4.05]). They shared accommodation or carpooled more frequently than the other cases. The outbreak investigation revealed a significantly increased risk of SARS-CoV-2 infection for workers of subcontractors and some foreign-born workers. There are many such populations in meat processing plants; the observed associations and the ways in which these workers are contaminated need to be confirmed by further work. Prevention campaigns should now target these workers. Environmental risk factors in the workplace setting remain to be clarified.

## Introduction

The COVID-19 outbreak due to SARS-CoV-2 was detected in China's Hubei province in December 2019 and spread rapidly around the world in early March 2020 (Bernard Stoecklin et al., 2020; Spiteri et al., 2020). To slow its spread in France, where the first cases were detected on 24 January, populations were first locked down from 17 March to 11 May 2020. After the lockdown, large-scale contact-tracing was implemented to quickly isolate contaminated people and then test and quarantine their contacts for 14 days. Contact-tracing was based on two secure digital platforms, one collecting the identities of people tested and the results of the laboratory tests (SI-DEP) and the other the identities of contacts (Contact-COVID).

On 13 May 2020, two days after the end of the lockdown, an intensive care unit from a French hospital informed the local health authorities of the admission of a person who tested positive for SARS-CoV-2 and worked in a pork and cattle processing plant. Following this report, 5 additional cases of COVID-19, confirmed since 09 May, were identified among the employees of the plant. They all worked in a same pork cutting workshop. Health authorities were aware of the presence of many non-French speaking workers who were difficult to investigate through contact-tracing.

Large outbreaks of COVID-19 in meat processing plants had already been reported in other countries, suggesting the presence of environmental and socio-economic risk factors specific to the meat processing plants (French National Academy of Medicine & the Veterinary Academy of France, 2020; Dyal et al., 2020; Middleton et al., 2020; Steinberg et al., 2020; Waltenburg et al., 2020). The main socio-demographic risk factors reported were the presence of a young workforce with few symptoms for COVID-19, the reliance of foreign-born workers housed and transported in crowded conditions, and job insecurity that discourages workers from reporting symptoms (Middleton et al., 2020).

Several works also suggest increased viral circulation among populations living near meat processing plants and highlight the public health issues associated with controlling COVID-19 clusters in these facilities (Middleton et al., 2020; Taylor et al., 2020).

Few cluster investigations are described in the scientific literature outside the USA and Germany. The article presents the investigation of the cluster in France in May 2020: it describes the workers infected with SARS-CoV-2 and assesses the associations with socio-demographic and occupational characteristics. The observed association measures will allow to implement risk management measures targeting workers at increased risk of contamination.

## Methods

### Data Collection

On the industrial site, the pork and cattle activities were carried out in two separate areas. The investigations were based on a cross-sectional study which was carried out in the pork section where the cluster occurred. In this area, 1,347 people were working at the time of the alert.

With the help of the company, workers’ activities were aggregated into four groups based on processing steps and similar environmental conditions. Primary pork processing corresponded to activities at ambient temperature: reception of animals, bleeding, evisceration, up to cold storage of half-carcasses (“Slaughter activities”). This was followed by the low-temperature (≤ 10 °C) deboning and cutting workshops and then the 3^rd^ processing activities producing sausages, rolled products and consumer sales units (“Transformation activities”). The 4^th^ group corresponded to the transverse functions: maintenance, upkeep, etc. To supplement its workforce, the company used temporary workers and subcontracting companies. Production was organised into 2 × 8-h shifts; cleaning was carried out at night on a third shift.

To break the chains of transmission, workers were tested by RT-PCR from nasopharyngeal swabs taken during four screening campaigns. Workers gave informed oral consent before being sampled. Laboratory sequencing activities were limited in May 2020 and no phylogenetic analysis was performed.

People diagnosed as positive for COVID-19 were isolated for a minimum of 8 to 10 days; their contacts were quarantined for 14 days.

A first campaign was performed on 15 May to test workers from the cutting workshop at the origin of the alert (A-workshop, *n* = 287). The campaign was repeated on 26 May for workers who tested negative on 15 May after excluding workers under quarantine.

A campaign carried out on 19 May extended screening to other workers (*n* = 993). These three campaigns (15, 19 and 26 May) were carried out on the industrial site. The fourth campaign concerned employees of a cleaning company: samples were taken on 25 May and the following days in a city laboratory (*n* = 67).

An occupational case was defined as any person who has worked in the pork section since 20 April with a positive RT-PCR test for COVID-19 between 9 and 30 May 2020. Occupational cases were identified from the results of the screening campaigns and by searching the SI-DEP platform.

A person who shared a confined space for at least 15 min with an occupational case or had face-to-face contact within one metre was defined as a contact person if he or she or the occupational case was not wearing a surgical or FFP2 mask at the time of contact.

Work contacts were contact persons working in the pork area. They were identified by employers and the occupational health service. Community contacts were contact persons who did not work in the pork section; they were identified by the contact-tracing teams.

Secondary community cases were community contacts who tested positive within 7 days after the contact. They were searched in the SI-DEP database from the list of community contacts.

Cases (occupational and secondary community) were described in terms of socio-demographic data (age, sex, place of birth), geographical distribution, clinical signs, and hospitalisations. Geographical distribution was assessed through distances between the centroid of the municipalities of residence and the meat plant. The presence of clinical signs at the time of sampling was obtained from the laboratories or, failing that, from SI-DEP. Hospital admissions, stays in intensive care units and deaths in hospital due to COVID-19 were searched by the health authorities in an information system for the follow-up of victims of exceptional health situations.

Occupational cases were also described in terms of employer, activity and working hours, and risky practices in the peri-occupational environment (carpooling, sharing accommodation). To limit potential bias due to non-French speaking workers, translators were present on-site for data collection and screening campaigns.

### Data Analysis

Associations between socio-demographic and occupational characteristics of cases and SARS-CoV-2 infection were investigated using a multivariable model.

The analyses were carried out in R version 3.6.1. Measures of association were assessed through risk ratios (RR). The attack rates and measures of association were modelled by Poisson regression. For each explanatory variable, levels associated with the lowest observed attack rates were selected as reference group. As the approach was exploratory, the explanatory variables were selected by a backward stepwise algorithm minimising the BIC criterion which is more parsimonious than the AIC one. Interaction terms were introduced after examining the correlation matrix of the model coefficients. The absence of over-dispersion of the model was validated.

## Results

### Study Population

The 1347 workers were predominantly male (62.7%) with a median age of 39 years (18–66).

The employer was known for 1339 workers: 35.2% (*n* = 471) were regular workers of the meat processing company, 37.9% (*n* = 507) were temporary workers, 23.7% (*n* = 318) were employees of subcontracting companies and 3.2% (*n* = 43) were employees of the veterinary administration. Nine subcontracting companies were identified: 48.6% of their employees worked in the deboning and cutting department and six companies provided deboning and cutting services.

Country of birth was documented for 1338 workers: 28.5% were foreign-born. Of the 1388 workers, 15.1% came from the same non-French speaking Eastern European country. In the rest of the text, the term "Eastern European workers" will be used to refer to this population.

The study population consisted of the 1179 workers tested (87.5% of workers). Tested workers were older with a median age of 40.0 years compared to 34.0 years for non-tested workers. Women were more present among the tested workers: sex ratio (M/F) of 1.6 compared to 2.3 for the non-tested workers. Getting tested was associated with the employer: 94.5% of the regular workers were tested, 86.4% of the temporary workers, 80.5% of the employees of the subcontracting companies and 74.4% of the employees of the veterinary administration.

Of the workers tested, 1054 (89.4%) were sampled during one screening campaign; 112 (9.5%) were sampled on more than one occasion (110 on two occasions, and two on three occasions). Of the workers tested repeatedly, 98 (87.5%) worked in the A-workshop where the alert originated.

### Occupational Cases

#### Descriptive Analyses

In total, 140 occupational cases were identified. The dates of the positive samples were between 09 and 28 May 2020. One hundred and thirteen (80.7%) had a positive RT-PCR during a screening campaign. The remaining 27 cases were identified through hospital or outpatient sampling.

Occupational cases had a median age of 41.0 years, similar to that of the study population. Men outnumbered women two to one. The median distance between the town of residence of the cases and the plant was 12.8 km (Table 1).

**Table 1:** Demographic, geographical and clinical characteristics of cases for a COVID-19 outbreak in a meat plant. France, 2020 (*N* = 155)

	Occupational cases (*n* = 140)	Secondary community cases (*n* = 15)
Sex ratio (M/F)	2.2	0.9
Median age (*min–max*)	41.0 (*19–62*)	22.2 (*1–57*)
Median distance in km between the town of residence and the meat plant (*min–max*)	12.8 (*1.9–65.9*)	1.9 (*1.9–22.1*)
Symptomatic at the time of sampling *n *(*%*)	24 (*17.1*)	1 (*8.3*)
Hospitalised *n* (%)	4 (*2.9*)	0 (*0.0*)
Intensive care unit admission *n* (%)	2 (*1.4*)	0 (*0.0*)
Died *n* (%)	0 (*0*)	0(*0.0*)

Of the 140 occupational cases, 24 (17.1%) were symptomatic at the time of sampling. No worker died from COVID-19. Four cases were hospitalised (2.9%), of which two were admitted to the intensive care unit (ICU). The mean age of the hospitalised cases was 43.2 years. They were all workers of the deboning and cutting department. The first case was hospitalised at the end of March, before the report, while the other three were hospitalised in May.

The two cases admitted to ICU were less than 50 years old. One of them was described through a non-exhaustive surveillance system for severe forms of COVID-19: he had no comorbidities as risk factors for severe COVID-19. The analysis of hospital data also identified three additional workers hospitalised with COVID-19 between the end of March and mid-April. These workers did not meet the case definition: two had not been tested and the third one had tested negative between 9 and 30 May 2020.

The majority of cases were employed by subcontracting companies (50.7%) or were temporary workers (30.7%). They mainly (56.8%) worked in the afternoon (Table 2).

**Table 2:** Univariate analysis for a COVID-19 outbreak in a meat plant. France, 2020 (*N* = 1179)

		All (%)	Occupational cases (*n* = 140) (%)	Non-cases (*n* = 1039) (%)	RR [95%CI]	p
Sex (*n* = 1178)	Men	727 (*61.7*)	96 (*68.6*)	631 (*60.8*)	1.35 [0.95 – 1.93]	0.096
	Female	451 (*38.3*)	44 (*31.4*)	407 (*39.2*)	Ref	
Age (*n* = 1179)	18–29 years	292 (*24.8*)	27 (*19.3*)	265 (*25.5*)	1.07^1^ [0.93 – 1.24]	0.34
	30–39 years	289 (*24.5*)	37 (*26.4*)	252 (*24.3*)		
	40–49 years	326 (*27.7*)	40 (*28.6*)	286 (*27.5*)		
	50–59 years	252 (*21.4*)	33 (*23.6*)	219 (*21.1*)		
	≥ 60 years	20 (*1.7*)	3 (*2.1*)	17 (*1.6*)		
Place of birth (*n* = 1171)	Foreign-born	335 (*28.6*)	73 (*52.1*)	262 (*25.4*)	2.72 [1.95 – 3.79]	< 10^–3^
	Born in France	836 (*71.4*)	67 (*47.9*)	769 (*74.6*)	Ref	
Place of birth (*n* = 1171)	Eastern European	187 (*16.0*)	63 (*45.0*)	124 (*12.0*)	4.31 [3.09 – 6.01]	< 10^–3^
	Other	984 (*84.0*)	77 (*55.0*)	907 (*88.0*)	Ref	
Native language (*n* = 1171)	Other	232 (19.8)	66 (47.1)	166 (16.1)	3.61 [2.59 – 5.03]	< 10^–3^
	French	939 (80.2)	74 (52.9)	865 (83.9)	Ref	
Employer (*n* = 1171)	Subcontractor	256 (*21.9*)	71 (*50.7*)	185 (*17.9*)	5.09 [3.25 – 7.97]	< 10^–3^
	Temporary workers	438 (*37.4*)	43 (*30.7*)	395 (*38.3*)	1.80 [1.11 – 2.93]	0.018
	Veterinary administration	32 (*2.7*)	0 (*0.0*)	32 (*3.1*)	Ref^2^	
	Regular workers	445 (*38.0*)	26 (*18.6*)	419 (*40.6*)		
Activity (*n* = 1172)	Deboning and cutting	637 (*54.3*)	114 (*81.4*)	523 (*50.7*)	3.68 [2.41 – 5.64]	< 10^–3^
	A-workshop	268 (*22.9*)	83 (*59.3*)	185 (*18.0*)		
	Primary cutting workshop	162 (*13.8*)	15 (*10.7*)	147 (*14.2*)		
	Other workshop	207 (*17.6*)	16 (*11.4*)	191 (*18.5*)		
	Primary processing	186 (*15.9*)	10 (*7.1*)	176 (*17.0*)	Ref^3^	
	3^rd^ processing	247 (*21.1*)	12 (*8.6*)	235 (*22.8*)		
	Transverse	102 (*8.7*)	4 (*2.9*)	98 (*9.5*)		
Work hours (*n* = 943)	Afternoon	351 (*37.2*)	71 (*56.8*)	280 (*34.2*)	3.72 [1.92 – 7.22]	< 10^–3^
	Morning	408 (*43.3*)	44 (*35.2*)	364 (*44.5*)	1.98 [1.00 – 3.94]	0.05
	Night	33 (*3.5*)	2 (*1.6*)	31 (*3.8*)	Ref^4^	
	Flexitime	137 (*14.5*)	8 (*6.4*)	129 (*15.8*)		
	In the day	14 (*1.5*)	0 (*0.0*)	14 (*1.7*)		

^1^RR provided for a 10-year increase in age

^2^Reference group consisted of regular workers of the meat processing company and workers of the veterinary administration

^3^Reference group consisted of workers outside the deboning and cutting department

^4^Reference group consisted of workers who work days, nights or flexitime

The attack rate in the study population was 11.9% (140/1179). It was 16.6% (114/687) among workers of the deboning and cutting department, reaching 28.9% (83/287) in the A-workshop at the origin of the report. In the primary cutting workshop, which had functional links with the A-workshop, an intermediate attack rate of 8.9% (15/167) was observed, compared with 6.9% (16/233) in the other cutting workshops. In total, workshops of the deboning and cutting department accounted for 81.4% of all cases (114/140) and the A-workshop for 59.3% (83/140).

In the A-workshop, the positivity rate was 31.4% (61 cases/194) for the first screening campaign organised on 15 May. Two additional workers who had not been tested in the first campaign tested positive after participating in the 19 May campaign targeting other workers. For the second campaign in the A-workshop (on 26 May), the positivity rate was 4.7% (4/86). The other 16 cases in the A-workshop were identified after outpatient sampling: two of them were detected after 26 May.

Attack rates were lower among other workers: 4.6% for workers of the primary processing activities, 4.1% for the 3rd processing activities, and 2.7% for transverse functions.

Foreign-born workers accounted for half of the cases (52.1%) compared to a quarter (25.4%) of non-cases (Table 2). Of the cases, 45.0% were Eastern European workers and 47.1% were non-French speakers. Of the 63 Eastern European cases, 60 (95.2%) worked in the deboning and cutting department and 42 (66.7%) were employed by subcontracting companies.

Contact-tracing forms were completed by 118 occupational cases (84.3%), 64 of whom were foreign-born. Sixty-two cases (52.5%) reported carpooling or sharing their accommodation with one or more other workers without specifying whether they were family members. Specifically, 49 cases (41.5%) reported carpooling to and from work with 1 to 4 other workers. And 40 cases (33.9%) reported sharing their accommodation with at least one other worker. Carpooling or sharing accommodation was more frequently reported by the Eastern European cases: 67.3% compared to 39.7% for the other cases (p = 5.10^–3^, Pearson's Chi2).

#### Association Measures

The variables used were documented for more than 99%, with the exception of work schedules, which were documented for 80.0% of the workers (Table 2). Timetables were documented for 70.6% of the regular workers  of the meat company and veterinary administration employees, 75% of the employees in the subcontracting companies and 94.5% of the temporary workers.

Two definitions of place of birth were explored for the selection of the multivariable model: foreign-born vs. born in France, or Eastern European worker vs. others. The second definition allowed to take into account the specificities of Eastern European workers compared to other foreign-born workers: a strong presence in the meat processing plant and local companies that could lead to a more active social life.

In univariate analysis, being an employee of a subcontracting company or an Eastern European worker were the 2 variables most associated with infection: the risk ratios were 5.09 [3.25—7.97] and 4.31 [3.09—6.01] respectively. To a lesser extent, associations were observed for foreign-born, non-French speaking, temporary, and afternoon shift workers (Table 2).

The multivariable Poisson regression was based on 1170 workers (99.2% of the workers tested). The selected variables were employer, activity in the company and place of birth (Eastern European worker vs. others).

Working for a subcontracting company was associated with the occurrence of SARS-CoV-2 infection: RR = 2.98 [1.81–4.99]. The modelled attack rate increased from 5.6% [3.7–8.3] in the reference group (regular and veterinary administration workers) to 16.5% [12.1–22.6] among workers in subcontracting companies (Table 3).

**Table 3:** Multivariable analyses for a COVID-19 outbreak in a meat plant. France, 2020 (*n* = 1170)

	Attack rate (%)	Risk ratio
Subcontractor	16.5 [12.1–22.6]	2.98 [1.81–4.99]
Temporary workers	7.4 [5.3–10.3]	1.34 [0.81–2.23]
Regular and veterinary administration workers	5.6 [3.7–8.3]	*Ref*
Eastern European workers in the deboning and cutting department	28.4 [20.3–39.8]	2.67 [1.76–4.05]
Other workers in the deboning and cutting department	10.6 [8.1–13.9]	*Ref*
Eastern European workers outside the deboning and cutting department	3.8 [1.2–12.1]	0.82 [0.19–2.38]
Other workers outside the deboning and cutting department	4.7 [3.1–7.1]	*Ref*

Outside the cutting department, Eastern European workers had the same risk of SARS-CoV-2 infection as other workers (RR = 0.82 [0.19–2.38]). In contrast, for the cutting department, the risk of infection was higher among Eastern workers with a risk ratio of 2.67 [1.76–4.05] (Table 3). For Eastern European workers, the modelled attack rate increased sharply in the case of cutting work from 3.8% [1.2–12.1] to 28.4% [20.3–39.8]. The increase in the attack rate was lower for other workers, from 4.7% [3.1–7.1] to 10.6% [8.1–13.9] (Table 3).

### Secondary Community Cases

A total of 368 community contacts were identified, ranging in age from 5 months to 86 years (median 32.9 years) and the sex ratio (M/F) was 0.8. RT-PCR results were recovered for 241 (65.5%) of these.

Fifteen secondary community cases (15/241 = 6.2%), diagnosed between 18 and 26 May, were identified. Eight were from two separate groupings: extended family (4 cases) and foreign-born workers in the beef Sect (4 cases).

The secondary community cases were younger than the occupational cases: 6 were under 18 years old and the median age was 22.2 years. The sex ratio (M/F) at 0.9 was more balanced (Table 1). The distance between the town of residence and the plant ranged from 1.9 to 22.1 km.

The health authorities collected the place of birth for 12 of the 15 secondary community cases: 8 (66.7%) were foreign-born.

Symptomatic character was documented in 12 secondary cases: 3 (25%) had been symptomatic in the 7 days prior to collection; only 1 (8.3%) was symptomatic at the time of collection (Table 1). No hospitalisations or deaths were identified.

## Discussion

The continuation of agri-food activities during the lockdown period favoured the occurrence of COVID-19 outbreaks in meat processing plants in several countries (Brazil, Canada, United States, France, Portugal, Italy, United Kingdom, etc.).

The scientific literature discusses the existence of socio-economic and environmental risk factors specific to these establishments and proposes prevention and control measures (French National Academy of Medicine & the Veterinary Academy of France, 2020; Middleton et al., 2020; Donaldson, 2020). The presence of metal surfaces, dense aerosol production, air handling maintaining low temperatures and high relative humidity, high noise levels requiring loud talking, and the density of workers on the production lines, are the main environmental factors discussed.

However, outbreak investigations in meat processing plants are poorly described outside Germany and the USA (Pokora et al., 2021; Dyal et al., 2020; Steinberg et al., 2020; Waltenburg et al., 2020; Donahue et al., 2020; Taylor et al., 2020). In France, surveillance of COVID-19 clusters identified the occurrence of 22 episodes representing at least 859 occupational cases between March and mid-September 2020. The results presented in the article correspond to the largest cluster (140 occupational cases) occurring in France in a meat processing plant that remained open throughout the lockdown in the first epidemic wave. These results complement the first descriptions of European outbreaks (Di Leone et al., 2020; Günther et al., 2020).

Among occupational cases, a prevalence of symptoms at the time of sampling of 17.1% was observed. Even if screening campaigns favour the detection of asymptomatic cases, the prevalence of symptoms is probably underestimated. This might be partly due to the large number of non-French speaking cases (47.1%) from whom the gathering of symptoms could be difficult. In comparison, clinical forms become more frequent with age, accounting for 21% (12–31%) of infections in 10–19 year-olds, rising to 69% (57–82%) in the over 70 s (Davies et al., 2020). As regards severe forms, the proportion of hospitalised cases (2.9%) remains consistent with French estimates: 2.9% (1.7–4.8%) of infections for all ages and 1.6% (0.9–2.6%) for 40–49 year-olds (Salje et al., 2020).

The investigations presented are based on screening of 87.5% of the workers. This high participation allows the main associations observed in the study population to be extrapolated with confidence to all workers.

The descriptive study revealed an outbreak confined to the deboning and cutting department, which accounted for 81.4% of cases. In meat processing plants, deboning and cutting workshops are conducive to the occurrence of COVID-19 clusters since they bring together the main environmental risk factors discussed in literature: lowest temperatures, high density of workers and low outdoor air flow per employee (Pokora et al., 2021). The workshop at the origin of the report alone accounted for 59.3% of the cases: the two screening campaigns and contact-tracing enabled the transmission chains to be rapidly controlled, since only four cases were detected during the second campaign, followed by two cases revealed after outpatient sampling.

At the time of the alert, the COVID-19 risk management measures in the plant were isolation of the symptomatic workers, wearing of surgical masks, and distancing rules at workstations and in common areas. Compliance with the measures was not assessed.

The epidemiological investigation of a COVID-19 outbreak in a meat processing plant does not, on its own, make it possible to validate the risk factors present in the environment of cutting activities (worker density, cold and confined atmosphere…) and assess the potential environmental routes of contamination (inhalation of airborne particles, contact with contaminated meat products or surfaces). Thus, no tests were performed to detect SARS-CoV-2 in meat products. Nevertheless, modelling work is envisaged to achieve these objectives after having gathered knowledge relating to the operation of the plants, the persistence and survival of the virus in the environment, and the epidemiological features of COVID-19 clusters.

The multivariable model showed an association between Eastern European workers and SARS-CoV-2 infection within the cutting department (RR = 2.67 [1.76–4.05]). This explanatory variable could be a proxy for specific housing conditions, means of commuting, and poor command of French. These all may affect adherence for social distancing or mask wearing within the company, in the peri-work environment or in the private sphere. As regards the peri-work environment, 67.3% of the cases among Eastern European workers reported carpooling or sharing accommodation, compared with 39.7% of the other cases. Similar practices among foreign-born workers have also been reported in the US poultry industry: workers were almost twice as likely to carpool (OR = 1.9) and share accommodation 6 times more (Rubenstein et al., 2020). The poor command in French, observed by the contact-tracing teams, could partly explain risky practices: partial understanding of health issues, barrier gestures and distancing measures.

A three-fold increase (RR = 2.98 [1.81–4.99]) in the risk of contamination was revealed for employees of the subcontracting companies. This association suggests the effect of specific practices within these companies. Specificities in terms of occupational health monitoring or management sensitivity to prevention and control measures would be worth exploring. Some symptomatic workers may also have continued to work in order not to lose work days due to the lack of compensation. Our results are consistent with other investigations of outbreaks in meat processing plants. For example, an investigation in the United States reported a cumulative attack rate of the disease 1.8 times higher among non-salaried employees of the meat plant (Steinberg et al., 2020). In addition, in Germany, Günther et al*.* refer to the significant presence of subcontractors on production lines (Günther et al., 2020).

The health authorities have not received any reports that would suggest a significant spread of SARS-CoV-2 in neighbouring populations, despite the fact that the occupational and secondary community cases were living in a restricted area around the company. This could be explained in part by the effectiveness of contact-tracing and by the presence of cases with a poor command of French and possibly little social contact with the local population.

Our study presents some limitations. First, the number of occupational cases may have been slightly underestimated by the non-exhaustive screening (87.5%) of workers. In the absence of serological testing, early infections may also have gone undetected. For example, one worker presented a negative RT-PCR after being hospitalised for COVID-19 between the end of March and mid-April. Undetected infections in the study population could reduce the strength of associations. Second, the transmission chains have not been described. Indeed, the dates of occurrence of the clinical signs and the workstations were not collected. The role of the first cases from March onwards in the dynamics of the episode cannot therefore be assessed and the hypothesis of a gradual spread of the outbreak from the A-workshop cannot be supported. The description of the transmission chains would also have complemented the discussion regarding contaminations attributable to peri-work practices (Günther et al., 2020). Third, compared to the other variables, there is more missing data on working hours and the data collected is a priori of lower quality. These three limitations regarding all categories of workers, seem however insufficient to question the selection of the model and the identification of populations at risk. And fourth, the number of secondary community cases is possibly underestimated. While the proportion of contacts with positive RT-PCR (6.2%) is consistent with the investigation of a cluster in the US (8.7%), misidentification of contacts, particularly from non-French speaking cases, cannot be excluded (Steinberg et al., 2020). In addition, screening results were only obtained for 65.5% of community contacts. This figure could reflect poor adherence to testing: some contacts may have already developed the disease and no longer see the point of testing. Finally, the digital contact-tracing platforms available from 13 May did not allow the identification of secondary cases that had been diagnosed earlier during the lockdown.

In conclusion, the investigation of the outbreak revealed a significantly increased risk of SARS-CoV-2 infection for workers of subcontractors and some foreign-born workers.

The observed associations and the ways in which workers are contaminated need to be confirmed by further work. However, these findings are consistent with the scientific literature and the strength of the observed associations should already be taken into account to prevent the occurrence of large clusters of COVID-19. Indeed, in the meat processing plants, foreign-born workers and workers from subcontractors are numerous. Particular attention must now be paid to their ability to appropriate and implement the COVID-19 risk management measures in terms of vaccination, early detection of symptoms, barrier gestures and distancing measures. Information and prevention messages must therefore be translated into the language of the workers. Messages must take into account the risks of contamination in the workplace, as well as in peri-professional and private life.

Representatives of the different populations could also be asked to facilitate the dissemination of prevention messages, promote vaccination and explain the method and objectives of contact-tracing especially when a cluster occurs. Implementation of vaccination campaigns within meat processing plants could also be an effective way to improve the vaccination coverage of workers.

## Data Availability

All data are present in the article or upon reasonable request from the corresponding author, although requests for data might require partial aggregation or down sampling to protect patient privacy. Source data are provided with this paper. The code is available upon any request.
